# Comparison among Different Scoring Systems in Predicting Procedural Success and Long-Term Outcomes after Percutaneous Coronary Intervention in Patients with Chronic Total Coronary Artery Occlusions

**DOI:** 10.3390/medicina55080494

**Published:** 2019-08-16

**Authors:** Artis Kalnins, Ieva Strele, Aivars Lejnieks

**Affiliations:** 1 Clinic of Cardiovascular diseases, Riga East University Hospital, Riga, LV 1038, Latvia; 2 Department of Public Health and Epidemiology, Riga Stradins University, Riga, LV 1007, Latvia; 3 Department of Internal Diseases, Faculty of Medicine, Riga Stradins University, Riga, LV 1007, Latvia

**Keywords:** chronic total occlusion, percutaneous coronary intervention, scoring systems

## Abstract

*Background and objectives:* Different scoring systems are used to stratify patients with chronic total coronary artery occlusions (CTO) according to disease complexity to predict the success of the percutaneous coronary intervention (PCI). Comparison among different CTO scoring systems and long-term outcome for patients with CTO after PCI has not been well established. The objectives of the study were to assess the ability of different disease severity scoring systems to predict, first, procedural success and, second, overall survival in patients with a successful procedure. *Materials and Methods:* A total of 551 patients who underwent elective CTO PCI in Riga East University hospital from January 2007 to December 2016 were included in the study. Four scoring systems (J CTO, PROGRESS CTO, CL, and CASTLE) were calculated. ROC curves were used to assess the association between scores and procedural success, and the Kaplan–Meier method and Cox regression were used to estimate the association with death from any cause after a successful procedure, *Results:* 454 of 551 cases were successful. With increasing disease complexity, the procedural success rate was significantly reduced in all scoring systems (*p* < 0.001): Area under the curve was 0.714 for J CTO score, 0.605 for PROGRESS CTO, 0.624 for CL and 0.641 for CASTLE scores. During the median 6.8 years of follow-up time, survival was better in the successful procedure group (*p* = 0.041). Among patients with procedural success, only PROGRESS and CASTLE scores showed an association with all-cause risk of death. After adjustment for baseline characteristics, patients having high PROGRESS score had almost twice higher risk of death (HR 1.81(95% CI 1.19–2.75)), and those with high and intermediate CASTLE score experienced almost four (HR 3.68(95% CI 1.50–9.05)) and two (HR 2.15, (95% CI 1.42–3.23)) times higher risk of death than the low score patients, respectively. *Conclusions:* All four CTO scoring systems had moderate ability to predict procedural success. More complex CTO PCI patients, assessed by PROGRESS and CASTLE scores, has worse all-cause survival in six to seven years after a successful procedure; whereas J CTO and CL scores had no association with survival.

## 1. Introduction

Percutaneous coronary interventions (PCI) for chronic total occlusions (CTO) remains one of the most difficult subsets for the interventional cardiologist because of the perceived procedural complexity. The widespread introduction of the latest CTO revascularization techniques, especially the retrograde approach [1,2], has made it possible to increase the number of successful procedures above 90%. The key to success in CTO PCI is diligent planning, which includes patient selection [3,4]. In selecting patients for CTO PCI it is important to consider patient-specific clinical and anatomical risk factors for the procedure [4]. Choosing the appropriate patients for CTO will maximize the benefits for the patient and minimize the risk of complications. To enhance procedural success, the pre-procedural case evaluation is important. Various scoring systems have been developed to evaluate CTO PCI procedural potential success. The most commonly used ones in the world are J CTO score (Multicenter CTO Registry in Japan) [4], the Prospective Global Registry for the Study of Chronic Total Occlusion Intervention score (PROGRESS CTO) [5], the clinical and lesion related score (CL) [6], and the coronary artery bypass grafting (CABG) history, age (≥70 y), stump anatomy (blunt or invisible), tortuosity degree (severe or unseen), length of occlusion (≥20 mm) and extent of calcification (severe) score (CASTLE) [7].

The concept of long-term outcome in interventional cardiology is not well defined. In most publications, the observation period ranges from one to five years, but there are studies, where it is up to 10 years [8].

Randomized studies do not prove a reduction in mortality after CTO recanalization [9,10]. Multiple observational studies have demonstrated the association of successful CTO revascularization with improved clinical outcomes compared to non-successful CTO revascularization: A meta-analysis of 25 studies compared successful (71%) with failed (29%) CTO PCIs in 28,486 patients. During a mean follow-up of 3.11 years, compared with failed procedures, successful CTO PCI was associated with lower mortality (odds ratio, 0.52), less residual angina (odds ratio, 0.38), lower risk of stroke (odds ratio, 0.72), and less need for subsequent CABG (odds ratio, 0.18) [8].

However, comparison of long-term results of successful and unsuccessful procedures has been reasonably criticized, because it is not a randomized comparison and it is likely that patients in whom CTO PCI fails have more complex angiographic characteristics and more comorbidities that can adversely affect subsequent outcomes [11]. Several clinical studies have also shown the relationship between the complexity of the procedure and procedural success [4,5,6,7]. The relationship between pre-procedural assessment results and the long-term outcome has been relatively poorly studied.

This study is a retrospective cohort single-center study, where we analyzed different scoring systems not only in predicting the procedural success but also in predicting long-term outcome (survival) after a successful CTO PCI procedure.

## 2. Materials and Methods

A total of 551 patients who underwent elective CTO PCI in Riga East University hospital from January 2007 to December 2016 were included in the study. These patients were stratified into two groups according to the CTO PCI procedure result: Successful cases group (*n* = 454) and unsuccessful cases group (*n* = 97). All CTO PCI procedures were performed by one operator. Patients’ medical history and symptoms were collected by physicians. All patients signed an informed consent on the interventional operation. The study was approved by the Riga Stradins University Ethics committee on 14 January 2010.

The J CTO score, PROGRESS CTO score, CL score, and CASTLE score were calculated retrospectively on the basis of coronary angiography and medical documentation. The primary endpoint was procedural success and all-cause death. CTO was defined as coronary complete occlusion (thrombolysis in myocardial infarction (MI) flow grade 0) with a duration of at least three months. The occlusion duration was estimated according to a previous history of myocardial infarction, the first onset of angina symptoms, or comparison with a previous angiogram [11,12].

If a single CTO lesion were attempted in two or more separate procedures during the enrolling period, only the last procedure was considered as a first and only attempt and subsequently included in the analysis. If two or more CTO PCIs were attempted for two or more separate CTO lesions during the enrolling period with a different result (one procedure successful, other not), the patient was excluded.

Procedural success was defined as the complete restoration of the antegrade blood flow (thrombolysis in MI flow grade 3) with an arterial lumen diameter reduction to less than 10% in the culprit CTO vessel.

### Statistical Analysis

IBM SPSS Statistics 22 software was used to perform statistical analysis. Categorical variables were summarized as proportions or percentages. Chi-square test or chi-square test for trend was used to assess the differences. Continuous variables, such as age, CL score, and follow-up time, were presented as mean values with standard deviations (SD) or median values with interquartile range (IQR). Student’s t-test was used to compare age and Mann–Whitney U test to compare score distributions between patients with successful and unsuccessful procedures.

Receiver operating characteristics (ROC) curves showing the area under the curve (AUC) were used to characterize the prognostic value of J CTO, PROGRESS, CASTLE, and CL scores in respect to procedure success.

Survival analysis was performed using Kaplan–Meier curves and Cox regression. Follow-up started at the date of PCI procedure and ended at the date of death or 15 April 2019, whichever came first. The endpoint was all-cause death. Log-rank test was used to compare Kaplan–Meier survival curves. Pairwise comparisons were made where necessary. Standard errors of survival proportions were used to estimate 95% confidence intervals (CI) and to calculate z-score to assess the difference in five-year survival by disease severity group. Mortality risk of patients with higher disease severity scores compared to the less severe group was estimated as hazard ratios (HR) measures using Cox regression model. They were adjusted for sex, age, and other baseline characteristics, such as smoking history, hypertension, dyslipidaemia, diabetes, history of MI, prior CABG, and prior PCI. The set of co-factors was specified for each model not to duplicate parameters that are already included in the specific disease severity score. *p* value below 0.05 was set as a value for statistical significance.

## 3. Results

Among 551 CTO PCI patients enrolled, 454 cases were successful. Mean age was 63.5 years and 80% were male. Forty-five percent had a history of coronary artery bypass grafting (CABG) and 52.5 had a prior PCI. Twenty percent of patients had diabetes, 72.6% had prior myocardial infarction (Table 1). We did not find significant differences between baseline characteristics in successful and unsuccessful cases group, except disease severity (Table 1) which was higher in the case of unsuccessful procedures, as would be expected. The distribution of patients according to the severity of the disease was uneven, with the majority of patients being in the low and intermediate difficulty group (Table 2).

**Figure 1:**
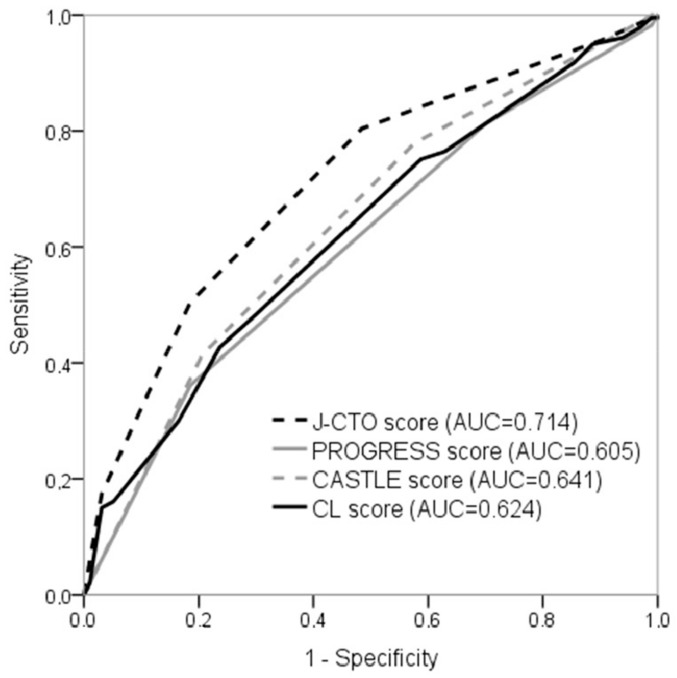
ROC curves: Prediction of procedure success by different scoring systems.

With increasing disease complexity, the procedural success rate was significantly reduced in all scoring systems. (Table 3). All four score systems showed a moderate predictive capacity (AUC for J CTO score 0.714, *p* < 0.001; AUC for PROGRESS CTO score 0.605, *p* < 0.001; AUC for CL score 0.624, *p* < 0.001; AUC for CASTLE score 0.641, *p* < 0.001) (Table 4). However, the J CTO score demonstrates an advantage over other scores (Figure 1).

**Figure 2:**
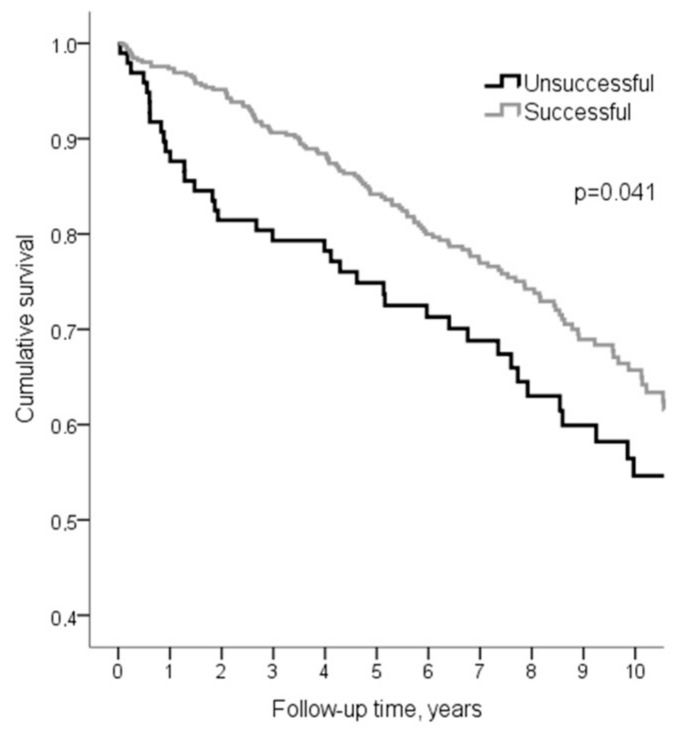
Survival after coronary artery occlusions percutaneous coronary intervention (CTO PCI). Successful and unsuccessful cases.

Median follow-up time was 6.8 years (IQR 4.1–9.3 years), the minimum and maximum follow-up was 1.2 years and 12.3 years, respectively. Statistical better survival was found in the successful procedure group (*p* = 0.041) (Figure 2).

**Figure 3:**
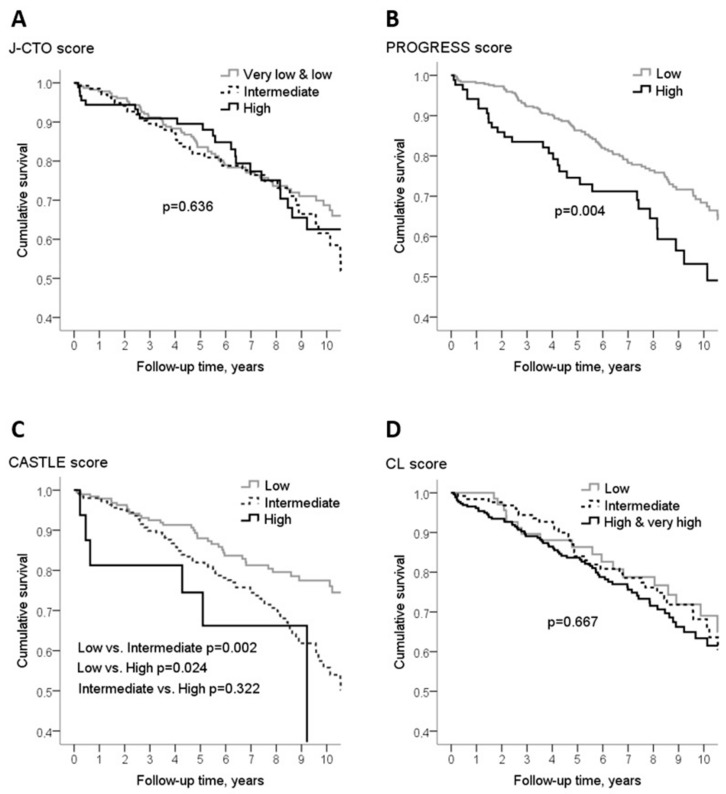
Survival of chronic total coronary artery occlusion patients after successful procedure by disease severity assessed using different scoring systems. (**A**) J CTO score, (**B**) PROGRESS score, (**C**) CASTLE score, (**D**) clinical and lesion related score (CL) score.

Disease severity assessed by PROGRESS score and CASTLE score showed an association with the risk of all-cause death (Figure 3) over the entire follow-up, however, five-year survival rate differed only in the case of PROGRESS score (Table 5). During the entire follow-up, patients having high PROGRESS score had almost two times higher risk of dying compared to the patients with low score (HR 1.81(95% CI 1.20–2.72)), and those with high and intermediate CASTLE score experienced almost three (HR 2.80(95% CI 1.18–6.66)) and two (HR 1.88(95%CI 1.26–2.78)) times higher risk of dying from any cause than the low score patients, respectively (Table 6). Adjustment for sex, age, and other baseline characteristics did not affect the estimates substantially (Table 6). The association between PROGRESS score and survival, after adjustment for age, sex, smoking history, hypertension, dyslipidemia, diabetes, prior MI, and prior PCI, was the same: HR 1.81(95%Cl 1.19–2.75). Whereas the association between CASTLE score and risk of all-cause death, adjusted for sex, smoking history, hypertension, dyslipidemia, diabetes, prior MI, prior PCI, and prior CABG, became even stronger: The risk of dying in patients with high CASTLE score was almost four times higher (HR3.68(95%Cl 1.50–9.05)) and in patients with intermediate score it was twice higher (HR 2.15(95%Cl 1.42–3.23)) compared to the patients with low CASTLE score.

## 4. Discussion

The present retrospective study analyses a cohort of 551 consecutive patients, who underwent the CTO-PCI procedure in one center over 10 years. Four hundred fifty-four procedures were successful. Our single-center study confirmed that patients after successful CTO PCI have a better long-term outcome in comparison with unsuccessful CTO PCI group. Several non-randomized comparative studies showed a beneficial effect of CTO recanalization on symptoms, quality of life, and left ventricular function [13,14], while its impact on survival remains unclear [12,13,14,15]. The effectiveness of CTO PCI to relieve symptoms has now been confirmed in Euro CTO trial. This trial showed a superior effect of PCI on angina frequency and quality of life, as compared with OMT, 12 months after randomization [16]. However, there is no evidence that successful CTO PCI can improve the long-term outcome. There was no difference in the incidence of major adverse cardiovascular events with CTO-PCI versus no CTO-PCI, but the study was limited by low power for clinical endpoints and high crossover rates between groups.

In the DECISION-CTO trial, the three-year rate of the composite endpoint of all-cause death, MI, stroke, and any revascularization in the intention-to-treat analysis was similar between the PCI and OMT groups (20.6% vs. 19.6%) [9].

CTO scoring systems were created to predict the result of the procedure [14]. Procedures with a higher CTO score are technically more complex and with less success rate.

As the first scoring system J CTO score was created in 2006. The system was designed to predict the possibility of CTO antegrade crossing with wire within 30 min [5]. Since 2006, coronary intervention techniques have changed and the J CTO scoring system has been criticized for containing subjective and biased factors. Several attempts have been made to create a better scoring system. Nevertheless, the prognostic value of the J CTO score remains high, which is also confirmed by our study. J CTO score contains mainly factors, that cannot in themselves worsen the patient prognosis (CTO stump, occluded segment length, occluded artery tortuosity, previous PCI attempt). This could be the reason, why it has less impact on long-term outcome than other scores.

This study was designed to ascertain whether the scoring results can predict the long-term outcome after successful CTO PCI procedure.

We did not find long-term survival differences among different complexity procedures, analyzing cases with J CTO and CL score systems. At the same time, analyzing cases with PROGERESS CTO score and CASTLE score, more complex patients had a statistically worse long-term prognosis. Some risk factors are included in several scoring systems but some are unique. PROGRESS CTO score differs from others as it is based on four angiographic only variables, among which there are collateral estimation and CTO localization in the left circumflex artery, they are not included in other scores. These two factors obviously make the procedure more difficult and the long-term results worsen. In terms of the maximal number of points, PROGRESS score has the smallest possible sum of points (only four) and patient apportionment is not as wide as in other scores. CASTLE score differs as it contains as risk factors patient age and myocardial infarction in the past. Aging is an independent predictor of adverse cardiovascular events [17,18].

The majority of patients (60%) with a CTO did not undergo previous MI [19].

Patients with a CTO and an implantable cardioverter defibrillator for primary or secondary prevention of sudden cardiac death have a higher incidence of appropriate delivered therapies and shocks as compared to patients with ischemic cardiomyopathy without a CTO [10]. A CTO in an infarct-related artery has been discovered as an independent predictor for the occurrence of ventricular arrhythmias, resulting in a two/three-fold higher recurrence rate, even after treatment with ablation [20,21]. It is possible that the necrotic zone and scar will act as an inducer of ventricular arrhythmias [20,21]. Perhaps, that is why patients with a higher CASTLE score have a worse long-term outcome.

Our study has some limitations. First, although all study patients received standard drug therapy, including Aspirin and P2Y12 receptor inhibitors, before PCI, pharmacotherapy during the follow-up period was not analyzed in this study. Second, the study includes CTO patients, treated with PCI from 2007 to 2016. Procedural techniques, experience, and success rates during the last decade have changed. Third, a lot of significant data (medications, comorbidities, procedural data) were not included in the analysis.

## 5. Conclusions

All four CTO scoring systems had moderate ability to predict procedural success. More complex CTO PCI patients assessed by PROGRESS and CASTLE scores had worse all-cause survival in six to seven years after a successful procedure, whereas J CTO and CL scores had no association with survival.

## Figures and Tables

**Table 1:** Patient baseline characteristics.

Variables	Total (*n* = 551)	Successful (*n* = 454)	Unsuccessful (*n* = 97)	*p* Value
Mean age (±SD)	63.5 (10.4)	63.3 (10.5)	64.3 (9.9)	0.394
Male, *n* (%)	441 (80.0%)	364 (80.2%)	77 (79.4%)	0.859
Smokers, *n* (%):				
Never	309 (56.1%)	248 (54.6%)	61 (62.9%)	0.296
Ex	173 (31.4%)	146 (32.2%)	27 (27.8%)	
Current	69 (12.5%)	60 (13.2%)	9 (9.3%)	
Hypertension, *n* (%)	461 (83.7%)	383 (84.4%)	78 (80.4%)	0.340
Dyslipidaemia, *n* (%)	390 (70.8%)	329 (72.5%)	61 (62.9%)	0.060
Diabetes, *n* (%)	110 (20.0%)	95 (20.9%)	15 (15.5%)	0.222
Prior MI, *n* (%)	400 (72.6%)	323 (71.1%)	77 (79.4%)	0.099
Prior CABG, *n* (%)	45 (8.2%)	35 (7.7%)	10 (10.3%)	0.396
Prior PCI, *n* (%)	289 (52.5%)	229 (50.4%)	60 (61.9%)	0.041
Severity score, median (IQR)				
J CTO	2 (1–3)	1 (1–2)	3 (2–3)	<0.001
PROGRESS	1 (0–1)	1 (0–1)	1 (1–2)	<0.001
CASTLE	2 (1–3)	2 (1–2)	2 (2–3)	<0.001
CL	3.5 (2.0–4.5)	3.5 (2.0–3.5)	3.5 (3.0–4.5)	<0.001

MI, myocardial infarction; CABG, coronary artery bypass grafting; PCI, percutaneous coronary intervention; IQR, interquartile range; J CTO, Multicenter CTO registry in Japan; PROGRESS, Prospective global registry for the study of chronic total occlusion intervention score; CASTLE, the coronary artery bypass grafting history, age (≥70 y), stump anatomy, tortuosity degree, length of occlusion and extent of calcification score; CL, clinical and lesion related score.

**Table 2:** Description of disease severity of patients according to different scoring systems.

J CTO		PROGRESS		CASTLE		CL Score	
Score	*n*	Score	*N*	Score	*N*		
0	82	0	182	0	62	Min	0
1	165	1	255	1	146	Max	8.0
2	165	2	105	2	202	Mean (SD)	3.2 (1.4)
3	111	3	9	3	118	Median (IQR)	3.5 (2.0–4.5)
4	28			4	22		

**Table 3:** Proportion of successful procedures according to disease severity assessed by different scoring systems.

Disease Severity (Score Values)	*n*	Successful Procedures, *n* (%)	*p* Value for Trend
**J CTO**			
Very low & low (0-1)	247	229 (92.7%)	<0.001
Intermediate (2)	165	136 (82.4%)	
High (3)	139	89 (64.0%)	
**PROGRESS**			
Low (0-1)	437	369 (84.4%)	0.014
High (2-3)	114	85 (74.6%)	
**CASTLE**			
Low (0-1)	208	188 (90.4%)	<0.001
Intermediate (2-3)	320	250 (78.1%)	
High (4)	23	16 (69.6%)	
**CL**			
Low (0–1)	71	68 (95.8%)	<0.001
Intermediate (1.5–2.5)	146	126 (86.3%)	
High & very high (3–8)	334	260 (77.8%)	

**Table 4:** ROC curves: Prediction of procedure success by different scoring systems.

Scoring System	AUC	95% CI	*p* Value
J CTO	0.714	0.660–0.768	<0.001
PROGRESS	0.605	0.546–0.665	0.001
CASTLE	0.641	0.581–0.701	<0.001
CL	0.624	0.565–0.683	<0.001

**Table 5:** Five-year survival rate by disease severity according to different scoring systems in patients with a successful procedure.

Disease Severity (Score Values)	5-Year Survival, %	95% CI, %	*p* Value
**J CTO**			
Very low & low (0-1)	83.5	78.4–88.6	NS
Intermediate (2)	81.9	75.2–88.6	
High (3)	89.5	83.0–96.0	
**PROGRESS**			
Low (0-1)	86.3	82.6–90.0	0.028
High (2-3)	74.6	64.8–84.4	
**CASTLE**			
Low (0-1)	88.0	83.1–92.9	NS
Intermediate (2-3)	81.9	77.0–86.8	
High (4)	74.5	52.9–96.1	
**CL**			
Low (0–1)	86.3	78.1–94.5	NS
Intermediate (1.5–2.5)	84.0	77.1–90.9	
High & very high (≥3)	83.7	79.0–88.4	

NS: *p* > 0.05. NS, non-significant.

**Table 6:** Association between disease severity and risk of all-cause death according to different scoring systems in patients after a successful procedure.

Scoring System	*N*	No. Deaths	Unadjusted			Model 1 *			Model 2 ^#^		
			HR	95% CI	*p* Value	HR	95% CI	*p* Value	HR	95% CI	*p* Value
**J CTO**											
Very low & low	229	59	Ref.			Ref.			Ref.		
Intermediate	136	38	1.22	0.81–1.83	0.344	1.21	0.80–1.82	0.359	1.22	0.80–1.85	0.350
High	89	23	1.11	0.68–1.79	0.685	1.03	0.63–1.67	0.911	1.12	0.67–1.86	0.670
**PROGRESS**											
Low	369	89	Ref.			Ref.			Ref.		
High	85	31	1.81	1.20–2.72	0.005	1.81	1.20–2.74	<0.001	1.81	1.19–2.75	0.005
**CASTLE**											
Low	188	37	Ref.			Ref.			Ref.		
Intermediate	250	77	1.88	1.26–2.78	0.002	1.90	1.28–2.82	0.001	2.15	1.42–3.23	<0.001
High	16	6	2.80	1.18–6.66	0.020	2.89	1.21–6.89	0.017	3.68	1.50–9.05	0.004
**CL**											
Low	68	19	Ref.			Ref.			Ref.		
Intermediate	126	33	1.02	0.58–1.79	0.947	1.03	0.58–1.81	0.928	1.04	0.59–1.85	0.893
High & very high	260	68	1.20	0.72–1.99	0.494	1.17	0.70–1.95	0.550	1.17	0.68–2.04	0.568

* Adjusted for age (except CASTLE and CL scores) and sex; ^#^ Adjusted for age (except CASTLE and CL scores), sex, smoking status, hypertension, dyslipidemia, diabetes, history of prior MI (except CL score), prior CABG (except PROGRESS and CASTLE scores), prior PCI, and procedure approach (retrograde or antegrade).
